# Postoperative delayed hyponatremia after endoscopic transsphenoidal surgery for pituitary adenomas: role of fluid restriction and associated risk factors

**DOI:** 10.3389/fendo.2026.1876280

**Published:** 2026-07-30

**Authors:** Aykut Gokbel, Ayse Uzuner, Eren Yilmaz, Atakan Emengen, Banu Kale, Sibel Balci, Savas Ceylan

**Affiliations:** 1 Department of Neurosurgery, Istinye University, Istanbul, Türkiye; 2 Department of Neurosurgery, Afsin State Hospital, Kahramanmaras, Türkiye; 3 Department of Neurosurgery, Bahcesehir University, Istanbul, Türkiye; 4 Department of Endocrinology, VM Medical Park Pendik Hospital, Istanbul, Türkiye; 5 Department of Biostatistics, Marmara University, Istanbul, Türkiye

**Keywords:** delayed postoperative hyponatremia, endoscopic transsphenoidal surgery, fluid restriction, hyponatremia, pituitary adenoma

## Abstract

**Background:**

Delayed postoperative hyponatremia remains a frequent complication after endoscopic endonasal transsphenoidal surgery (EETS) for pituitary adenomas and is a major cause of hospital readmission. Although fluid restriction has emerged as a potential preventive strategy, evidence regarding its efficacy and associated predictive factors remains limited. This study aimed to evaluate the impact of a standardized postoperative fluid-restriction protocol on the incidence of postoperative delayed hyponatremia and to identify factors associated with postoperative delayed hyponatremia development.

**Methods:**

A retrospective cohort study was conducted including 222 patients who underwent EETS for pituitary adenomas between January 2025 and January 2026. Patients with preoperative diabetes insipidus or requiring postoperative desmopressin treatment were excluded. Patients treated before implementation of routine postoperative fluid restriction constituted the non–fluid-restricted group (n=120), whereas patients treated after protocol implementation received fluid restriction of 1000 mL/day for 7 postoperative days (n=102). Delayed postoperative hyponatremia was defined as serum sodium <135 mmol/L occurring after postoperative day 3. Clinical, radiological, endocrine, and biochemical variables were analyzed.

**Results:**

Delayed postoperative hyponatremia developed in 30 patients (13.5%). The incidence was significantly lower in the fluid-restricted group than in the non–fluid-restricted group (6.9% vs 19.2%, p=0.013). Mean onset of delayed postoperative hyponatremia was postoperative day 7.9, and 93.3% of affected patients required readmission. Delayed postoperative hyponatremia development showed no significant association with age (p=0.963), sex (p=0.810), body mass index (p=0.446), tumor diameter (p=0.505), Knosp grade (p=0.350), extent of resection (p=0.289), adenoma subtype (p=0.267), or remission status (p=0.735). In contrast, suprasellar extension (p=0.010), postoperative pituitary hormone deficiency (p=0.001), preoperative hypothyroidism (p=0.033), diaphragma sellae descent (p<0.001), lumbar drainage (p<0.001), and sodium decline ≥3 mEq/L (p=0.002) were significantly associated with delayed postoperative hyponatremia. Multivariate logistic regression identified preoperative comorbidities (p=0.002), postoperative diaphragma sellae descent (p=0.029), postoperative hypothyroidism (p=0.011), postoperative hypokalemia (p=0.002), and longer hospital stay (p<0.001) as independent predictors of delayed postoperative hyponatremia.

**Conclusions:**

Routine postoperative fluid restriction significantly reduced the incidence of delayed postoperative hyponatremia following EETS for pituitary adenomas. Early postoperative sodium changes and selected perioperative factors may help identify high-risk patients and improve postoperative monitoring strategies.

## 1 Introduction

Endoscopic endonasal transsphenoidal surgery (EETS) is currently the preferred surgical approach for treating pituitary adenomas and other sellar pathologies. Although advances in surgical techniques have reduced morbidity, postoperative electrolyte disturbances remain a common clinical challenge, with hyponatremia being one of the most common complications.

Delayed hyponatremia is typically defined as a serum sodium concentration below 135 mEq/L occurring after the third postoperative day, with incidence rates ranging from 8% to 35% (1–9). This condition usually manifests between postoperative days 4 and 10, often reaching its nadir around the end of the first postoperative week (1–3, 10). While mild decreases in sodium levels may remain asymptomatic or present nonspecific symptoms, more drastic decreases can result in serious neurological complications, including altered consciousness, seizures, and cerebral edema (8, 9, 11, 12). Notably, delayed hyponatremia represents a major cause of unplanned readmissions following EETS and contributes substantially to postoperative morbidity (2, 9, 13).

Syndrome of inappropriate antidiuretic hormone secretion (SIADH) is considered the principal presumed pathophysiological mechanism underlying isolated delayed postoperative hyponatremia after pituitary surgery, which typically develops around postoperative days 6–8. However, delayed postoperative hyponatremia may also occur as the antidiuretic/SIADH-like phase of a biphasic or triphasic water-balance disorder. Disruption or irritation of the hypothalamic–neurohypophyseal axis during surgical manipulation may lead to dysregulated release of antidiuretic hormone, resulting either in isolated delayed postoperative hyponatremia or, in some patients, as part of a biphasic or triphasic response following pituitary surgery. The classic triphasic response consists of an initial diabetes insipidus/arginine vasopressin deficiency phase, followed by an antidiuretic hyponatremic phase, and finally recurrent diabetes insipidus. This distinction is clinically important because isolated delayed postoperative hyponatremia and delayed hyponatremia occurring as part of a triphasic response differ in their pathophysiology and prognostic implications, particularly regarding the risk of persistent diabetes insipidus. Other potential etiologies of delayed postoperative hyponatremia include cerebral salt wasting, adrenal insufficiency, and hypothyroidism; however, their relative contributions are less clear, and differentiation in the clinical setting may be challenging (6, 14–17).

Minimally invasive endoscopic approaches have driven a transition toward shorter postoperative hospitalization, which introduces an additional concern because many patients develop hyponatremia after discharge. This delayed presentation may hinder early recognition and timely intervention, increasing the likelihood of symptomatic deterioration and rehospitalization (18). While management approaches for delayed postoperative hyponatremia are well-established—primarily involving fluid restriction and close biochemical monitoring—preventive strategies are less clearly defined. Previous studies have attempted to identify prognostic factors for postoperative hyponatremia, but results have been inconsistent, probably due to variability in patient populations, surgical techniques, and postoperative management protocols. Furthermore, no universally accepted risk model has been established (4, 6, 19). In this context, postoperative fluid management protocols, particularly routine fluid restriction, have emerged as a potential preventive measure. Although some studies have demonstrated the beneficial effects of fluid restriction, the existing evidence is limited by heterogeneity in study design and patient selection (20–24).

In light of these considerations, further investigation is warranted to better define preventive strategies and identify patients at risk. Therefore, the present study has two objectives: 1) to evaluate whether a standardized postoperative fluid-restriction protocol reduces the incidence of delayed postoperative hyponatremia in patients undergoing EETS for pituitary adenoma and 2) to identify associated risk factors.

## 2 Materials and methods

### 2.1 Study design and patient population

This retrospective cohort study was conducted at a single tertiary center following institutional review board approval. Due to the retrospective design, the requirement for informed consent was waived.

The study included adult patients who underwent EETS for histopathologically confirmed pituitary adenomas, including patients presenting with pituitary apoplexy. To maintain cohort homogeneity, patients with other sellar or parasellar lesions were excluded. Additionally, patients with preoperative diabetes insipidus (DI) and those requiring desmopressin in the early postoperative period were excluded from the analysis. Patients with prolactinomas were considered for surgical treatment only after multidisciplinary evaluation. Indications for surgery included dopamine agonist resistance, dopamine agonist intolerance, visual deterioration or symptomatic mass effect, pituitary apoplexy, cystic or hemorrhagic tumor morphology, and patient preference following detailed discussion of available treatment options.

Patients with pituitary apoplexy were included according to our institutional treatment protocol. All patients with pituitary apoplexy routinely received perioperative glucocorticoid replacement therapy and underwent comprehensive endocrine evaluation. Therefore, untreated adrenal insufficiency or hypothyroidism was considered unlikely to represent the primary cause of delayed postoperative hyponatremia in this subgroup.

### 2.2 Group allocation

Patients were divided into two groups according to postoperative fluid management. A standardized postoperative fluid-restriction protocol was introduced in September 2025. Therefore, patients who underwent surgery between January and August 2025 constituted the non–fluid-restricted cohort, whereas patients treated between September 2025 and January 2026 comprised the fluid-restricted cohort. Thus, the study used a retrospective before-and-after cohort design based on the date of protocol implementation. In the latter group, fluid restriction was implemented as a preventive postoperative strategy. Oral fluid intake was routinely limited to 1000 mL/day, beginning on postoperative day 1, continued throughout hospitalization, and maintained after discharge to complete a total of 7 postoperative days unless contraindicated. Patients discharged before postoperative day 7 were instructed to continue the same 1000 mL/day limit at home until completion of the 7-day postoperative period. The fixed 1000-mL/day target was used as a pragmatic, standardized prophylactic limit rather than as a weight-based maintenance-fluid calculation. This volume was consistent with several previous postoperative transsphenoidal protocols using 1.0 L/day (20, 22). The protocol was not applied to patients with postoperative diabetes insipidus, hypernatremia, renal insufficiency, or congestive heart failure, in whom individualized postoperative fluid management was considered more appropriate.

### 2.3 Definition of delayed postoperative hyponatremia

Delayed postoperative hyponatremia was defined as serum sodium <135 mmol/L occurring after postoperative day 3, regardless of symptom status. Compatible symptoms, including nausea, vomiting, headache, fatigue, or altered mental status, were recorded when present (8). Alternative causes of delayed postoperative hyponatremia, including adrenal insufficiency and hypothyroidism, were evaluated using postoperative endocrine assessment and laboratory findings available in the medical records. Available clinical documentation related to extracellular volume status was reviewed, and cerebral salt wasting was clinically considered in patients with findings suggestive of hypovolemia. Available medication history, including recent diuretic exposure, was also reviewed when documented. However, because complete diagnostic criteria for SIADH—including serum/plasma osmolality, urine osmolality, urine sodium concentration, standardized clinical assessment and documentation of extracellular volume status, dietary sodium intake, and confirmation of no recent diuretic use—were not consistently available in this retrospective cohort, the primary outcome was defined as delayed postoperative hyponatremia rather than confirmed SIADH. Definitive differentiation between SIADH and other etiologies could therefore not always be established. In patients with pituitary apoplexy, perioperative glucocorticoid replacement therapy was routinely administered according to institutional practice.

### 2.4 Data collection

Clinical, radiological, and laboratory data were retrospectively collected from electronic medical records.

Demographic variables, including age, sex, and body mass index (BMI), and clinical characteristics, including comorbidities (hypertension, diabetes mellitus, cardiac and renal disease), tumor pathology, and history of previous surgery, were also recorded.

Radiological parameters, including maximum tumor diameter, suprasellar extension, cavernous sinus invasion, and Knosp classification, were assessed. Suprasellar extension was defined as superior tumor extension beyond the diaphragma sellae into the suprasellar cistern on preoperative MRI. In this retrospective dataset, suprasellar extension was recorded as a binary radiological variable (present or absent) and was not further graded according to the degree of superior extension. Postoperative diaphragma sellae descent was defined as downward displacement or collapse of the diaphragma sellae into the sellar cavity on postoperative imaging after tumor removal. It was recorded as a separate binary postoperative radiological variable and was not considered equivalent to preoperative suprasellar extension.

Tumors were classified as microadenomas (<10 mm) or macroadenomas (≥10 mm) according to the maximum tumor diameter measured on preoperative MRI. Giant adenomas were defined as lesions measuring ≥40 mm.

Surgical and postoperative radiological variables included extent of resection, postoperative diaphragma sellae descent, and tumor consistency (firm vs. soft). Extent of resection was determined according to the early postoperative MRI findings. Gross-total resection (GTR) was defined as complete tumor removal, with no visible residual tumor on early postoperative MRI. Near-total resection (NTR) was defined as resection of ≥95% but <100% of the tumor. Subtotal resection (STR) was defined as resection of >70% but <95% of the tumor.

Preoperative and postoperative laboratory parameters were extensively analyzed, including serum sodium, potassium, and glucose levels. Additionally, endocrine parameters such as pre- and postoperative hormonal deficiencies, hypocortisolism, and thyroid function abnormalities were evaluated.

Thyroid function was assessed preoperatively and routinely on postoperative day 1. The postoperative hypothyroidism variable used in the analysis was defined according to a postoperative day 1 biochemical thyroid profile compatible with central hypothyroidism, based on the institutional endocrinology protocol. This variable reflected early postoperative biochemical thyroid status rather than the presence or adequacy of thyroid hormone replacement. In patients who were not receiving levothyroxine preoperatively, additional endocrine evaluation was performed and thyroid hormone replacement was prescribed when indicated. In patients already receiving levothyroxine, the postoperative treatment regimen was reviewed by the endocrinology team and the dose was modified when considered necessary. Because delayed postoperative hyponatremia was defined as serum sodium <135 mmol/L occurring after postoperative day 3, the postoperative day 1 thyroid assessment preceded the qualifying sodium decline.

Postoperative variables, including length of hospital stay, lumbar drainage usage, remission status for secretory adenomas, and follow-up duration, were analyzed.

### 2.5 Peroperative management

All patients underwent standardized preoperative endocrine evaluation. Serum sodium levels were routinely monitored preoperatively and on postoperative day 1, with additional measurements performed in symptomatic patients.

Fluid intake and urine output were closely monitored during the postoperative period. Patients were discharged after clinical stabilization and normalization of electrolyte levels, and outpatient follow-up, including sodium monitoring, was recommended in select cases.

Fluid restriction was implemented as a preventive postoperative strategy rather than as treatment for established delayed postoperative hyponatremia. The protocol was not applied to patients who developed postoperative diabetes insipidus or hypernatremia, or to those with renal insufficiency or congestive heart failure, in whom individualized postoperative fluid management was considered more appropriate. Patients and their caregivers were informed that, if postoperative diabetes insipidus/arginine vasopressin deficiency (AVP-D), hypernatremia, clinically significant hypovolemia, worsening renal function, or any other safety concern developed after initiation of fluid restriction, the protocol would be discontinued or liberalized. During hospitalization, oral fluid intake and urine output were monitored through routine intake–output records, allowing assessment of adherence while patients remained in the hospital. After discharge, patients were instructed to continue the prescribed 1000-mL/day restriction until completion of the seventh postoperative day. However, no standardized fluid diary, daily intake log, or quantitative adherence instrument was used, and adherence was not systematically verified during outpatient follow-up. Therefore, postdischarge adherence could not be objectively quantified.

### 2.6 Outcome measures

The primary outcome was the incidence of delayed postoperative hyponatremia. Secondary outcomes included delayed postoperative hyponatremia -related readmissions and identification of risk factors associated with delayed postoperative hyponatremia development.

### 2.7 Statistical analysis

All statistical analyses were performed using IBM SPSS version 29.0 (IBM Corp., Armonk, NY, USA) and MedCalc 14 (MedCalc Software, Ostend, Belgium). The Kolmogorov-Smirnov and Shapiro-Wilk tests were used to assess the normality of the data. Continuous variables were presented as median with interquartile range (IQR) since the normality assumption did not hold. Categorical variables were summarized as counts and percentages. Between-group comparisons were conducted using the Mann-Whitney U test. Associations between categorical variables were analyzed using the Chi-square test. Binary logistic regression analysis was performed for multivariate evaluation. Receiver-operating characteristic (ROC) analysis was performed to compute the area under the curve (AUROC), sensitivity, specificity, and cut-off values. The absolute risk reduction was calculated as the difference in the observed incidence of delayed postoperative hyponatremia between the groups. The number needed to treat was calculated as the reciprocal of the absolute risk reduction and was rounded up to the next whole number. Because this was a retrospective before-and-after cohort study, the NNT was considered a descriptive estimate based on the unadjusted observed event rates. A p-value <0.05 was considered statistically significant.

## 3 Results

### 3.1 Patient cohort and demographics

Between January 2025 and January 2026, 351 patients underwent EETS for resection of pituitary adenomas, including cases presenting with pituitary apoplexy, performed by the senior author (S.C.) at VM Medical Park Pendik Hospital, Istanbul, Turkey. A total of 7 and 122 patients were excluded from the study due to a diagnosis of DI requiring desmopressin treatment in the preoperative and acute postoperative periods, respectively, resulting in a final cohort of 222 patients, whose characteristics are summarized in **Table 1**.

**Table 1:** Baseline characteristics of the study population and comparison of characteristics between patients who developed delayed postoperative hyponatremia and those who did not. Quantitative variables are expressed as mean (range) and median (interquartile range), while qualitative variables are presented as n (%). IQR, interquartile range.

	TOTAL			DELAYED POSTOPERATIVE HYPONATREMIA			NON-DELAYED POSTOPERATIVE HYPONATREMIA			
Characteristics	Mean (Range)	Median (IQR)	%	Mean (Range)	Median (IQR)	%	Mean (Range)	Median (IQR)	%	p
Patients, n	222		100	30			192			
Age at surgery, years	39.42 (18–73)	37 (31–50)		39.43 (20–64)	37.5 (31–48.5)		39.42 (18–73)	37 (31–50)		0.963
Sex										
Female	125		56.3	18		60	107		55.7	0.81
Male	97		43.7	12		40	85		44.3	
Comorbidities										
Yes	82		36.9	22		73.3	60		31.3	<0.001
No	140		63.1	8		26.7	132		68.7	
History of Prior Surgery										
Yes	55		24.8	9		30	46		24	0.627
No	167		75.2	21		70	146		76	
Preoperative Hormonal Deficiency (at least one axis)										
Yes (n=88)	88		39.6	16		53.3	72		37.5	0.148
Hypercortisolism	77/222		34.7	Nov-77		36.7	66/77		34.4	0.969
Hypothyroidism	28/222		12.6	Aug-28		26.7	20/28		10.4	0.033
Body Mass Index (BMI) (kg/m²)	28.04 (17.47–43.71)	26.92 (23.97–32.02)		26.67 (20.34–33.20)	26.21 (23.91–29.76)		28.25 (17.47–43.71)	27 (23.91–32.10)		0.446
Maximum Tumor Diameter (mm)	20.73 (6–60)	20 (13–27)		22 (7–40)	19 (11.75–33)		20.54 (6–60)	20 (13–27)		0.505
Preoperative Sodium Level (mEq/L)	139.47 (134–145)	140 (138–141)		138.60 (137–142)	139 (137–139)		139.60 (134–145)	140 (138–141)		0.005
Postoperative Day 1 Sodium Level (mEq/L)	138.44 (130–144)	139 (137–140)		135.37 (130–139)	135 (135–138)		138.92 (135–144)	139 (137.25–140)		<0.001
Preoperative–Postoperative Sodium Change (mEq/L)	1.91 (0–8)	1.5 (1–3)								0.002
<3 (mEq/L)	166		74.8	15		50	151		78.6	
≥3 (mEq/L)	56		25.2	15		50	41		21.4	
Preoperative Glucose Level (mg/dL)	102.86 (80–150)	100 (90–110.25)		104.83 (80–150)	102 (90–102)		102.55 (80–141)	99 (90–111)		0.946
Postoperative Day 1 Glucose Level (mg/dL)	121.36 (79–200)	119 (106.5–132)		114.23 (79–150)	117.5 (108–127)		122.47 (80–200)	119 (105–132)		0.208
Preoperative Potassium Level (mEq/L)	4.28 (3.5–5.2)	4.2 (4.1–4.5)		4.16 (3.8–4.4)	4.2 (4.1–4.3)		4.3 (3.5–5.2)	4.2 (4.1–4.6)		0.202
Postoperative Day 1 Potassium Level (mEq/L)	4.17 (3.2–5.4)	4.2 (4–4.4)		4.05 (3.2–5.4)	4 (3.4–4.4)		4.19 (3.2–5.2)	4.2 (4–4.4)		0.089
Postoperative Hypokalemia (Potassium Level<3.5 mEq/L)										
Yes	30		13.5	12		40	18		9.4	<0.001
No	192		86.5	18		60	164		90.6	
Suprasellar Extension of Tumor										
Yes	57		25.7	17		56.7	40		20.8	0.01
No	165		74.3	13		43.3	152		79.2	
Knosp Grading										
<3 (Grades 0, 1 and 2)	166		74.8	5		16.7	161		83.9	0.35
≥3 (Grades 3 and 4)	56		25.2	25		83.3	31		16.1	
Adenoma Type										
Non-Secretory and Apoplexy	106		47.7	11		36.7	95		49.5	0.267
Secretory	116		52.3	19		63.3	97		50.5	
Ki-67 Index	2.64 (1–12)	2 (2–3)								1
<3%	166		74.8	20		66.7	128		66.7	
≥3%	56		25.2	10		33.3	64		33.3	
Diaphragma Sellae Descent										
Yes	84		37.8	22		73.3	62		32.3	<0.001
No	138		62.2	8		26.7	130		67.7	
Postoperative Lumbar Drainage										
Yes	34		15.3	15		15.3	19		9.9	<0.001
No	188		84.7	15		84.7	173		90.1	
Multilayer Closure Technique										
Yes	72		32.4	14		46.7	58		30.2	0.114
No	150		67.6	16		53.3	134		69.8	
Fat Graft to the Sellar Area										
Yes	54		24.3	11		36.7	43		22.4	0.143
No	168		75.7	19		63.3	149		77.6	
Resection										
Gross Total (GTR)	172		77.5	26		86.7	146		76	0.289
Other than GTR	50		22.5	4		13.3	46		24	
Biochemical Remission Status of Secretory Adenomas (n=116)										
Yes	99		85.3	17		89.5	82		84.5	0.735
No	17		14.7	2		10.5	15		15.5	
Postoperative Hormonal Deficiency (at least one axis)										
Yes (n=89)	89		40.1	21		70	68		35.4	0.001
Hypercortisolism	67/222		30.2	16/67		53.3	51/67		26.6	0.006
Hypothyroidism	46/222		20.7	14/32		46.7	46/32		16.7	<0.001
Length of Hospital Stay (days)	2.84 (2–8)	2 (2–3)		4.63 (2–8)	5 (3–6.25)		2.56 (2–6)	2 (2–3)		0.001

The average age of the cohort was 39.42 ± 11.94 (range, 18–73) years, and the cohort comprised 125 women (56.3%) and 97 men (43.7%).

The non–fluid-restricted group (Group 1) comprised 120 patients treated between January and August, 2025; the fluid-restricted group (Group 2) comprised 102 patients treated between September 2025 and January 2026.

### 3.2 Preoperative clinical and radiological characteristics

The preoperative clinical and radiological characteristics of the cohort are presented in **Table 1**, while some comparative findings between Groups 1 and 2 are presented in **Table 2**.

**Table 2:** Characteristics of Group 1 (non–fluid-restricted) and Group 2 (fluid-restricted), including comparisons for the overall cohort and for patients who developed postoperative delayed hyponatremia. ARR, absolute risk reduction; NNT, number needed to treat. NNT was calculated as 1/ARR and rounded up to the next whole number. Quantitative variables are expressed as mean (range) and median (interquartile range), while qualitative variables are expressed as n (%). IQR, interquartile range.

	Group 1 (FluidUnrestricted)			Group 2 (FluidRestricted)			
Characteristics	Mean (Range)	Median (IQR)	%	Mean (Range)	Median (IQR)	%	p
Patients, n	120		54.1	102		45.9	
Delayed Postoperative Hyponatremia							0.013
Yes	23		19.2	7		6.9	
No	97		80.8	95		93.1	
Absolute Risk Reduction		12.3 percentage points					
Estimated number needed to treat		9					0.013
Age at surgery, years	38.85 (20–73)	37 (31–47.75)		40 (18–73)	37 (31.75–51)		0.529
Sex							
Female	68		56.7	57		55.9	1
Male	52		43.3	45		44.1	
Comorbidities							
Yes	49		40.8	33		32.3	0.211
No	71		59.2	69		67.7	
Body Mass Index (BMI) (kg/m²)	27.65 (17.47–43.71)	25.76 (24.05–30.75)		28.50 (18.18–43.71)	27.23 (23.83–32.33)		0.339
<25 (kg/m²)	51		42.5	37		36.3	0.409
≥25 (kg/m²)	69		57.5	65		63.7	
Postoperative Day 1 Sodium Level (mEq/L)	138.28 (130–143)	139 (137–140)		138.62 (130–144)	139 (137–140)		0.441
Preoperative–Postoperative Sodium Change Value (mEq/L)	1.99 (0–8)	2 (1–3)		1.81 (0–8)	1 (1–2)		0.503
<3 (mEq/L)	88		73.3	78		76.5	0.643
≥3 (mEq/L)	32		26.7	24		23.5	
Mean Delayed Postoperative Na Value, mEq/L	124.7 (112–134)	126 (120–129)		125.3 (118–129)	126 (120–129)		0.92
Mild Hyponatremia (131–135 mEq/L), n	Feb-23		8.7	0/7		0	0.557
Moderate Hyponatremia (126–130 mEq/L), n	Dec-23		52.2	05-Jul		71.4	
Severe Hyponatremia (≤125 mEq/L), n	Sep-23		39.1	02-Jul		28.6	
Mean Onset of Delayed Postoperative Hyponatremia, days	8 (6–12)	8 (7–9)		7.3 (6–10)	7 (6–9)		0.293
Symptomatic (Re-admission)	22/23		95.7	06-Jul		85.7	0.418
Yes	Jan-23		4.3	01-Jul		14.3	
No							
Hospital Readmission	21/23		91.3	07-Jul		100	1
Yes	Feb-23		8.7	0/7		0	
No							
Length of Hospital Stay for Readmission, days	3.71 (3–5)	4 (3–4)		5 (3–8)	5 (4–6)		0.153

A total of 82 patients (36.9%) had at least one comorbidity and were on medical treatment for the same. The most prevalent comorbidity was diabetes mellitus (59/222 patients, 26.6%), followed by hypertension (41/222, 18.5%) and hypercholesterolemia (19/222, 8.6%). Nephrological pathologies were present in 4/222 patients (1.8%), cardiac comorbidities and malignancy in 3/222 patients each (1.4%), and other comorbidities in 5/222 patients (2.3%).

A total of 55 patients (24.8%) had a history of surgery. Preoperatively, hormonal deficiency involving at least one axis was present in 88 patients (39.6%), including hypocortisolism in 77 (34.7%) and hypothyroidism in 28 (12.6%). The mean BMI of the cohort was 28.04 ± 6.01 (range, 17.47–43.71) kg/m². The mean maximum tumor diameter was 20.73 ± 10.08 (range, 6–60) mm. Based on maximum tumor diameter, 33 lesions (14.9%) were classified as microadenomas (<10 mm), whereas 189 (85.1%) were macroadenomas (≥10 mm). Among the macroadenomas, five lesions met the definition of giant adenoma (≥40 mm). Preoperative suprasellar extension of the lesion was observed in 57 patients (25.7%). According to Knosp grading, 29 patients (13.1%) were grade 0, 81 (36.5%) were grade 1, 56 (25.2%) were grade 2, 42 (18.9%) were grade 3, and 14 (6.3%) were grade 4.

No significant differences were observed between Groups 1 and 2 in terms of history of surgery (23.3% vs. 26.5%, p = 0.641), BMI (27.65 ± 5.91 vs. 28.51 ± 6.12 kg/m², p = 0.339), preoperative maximum tumor diameter (20.64 ± 10.11 vs. 20.84 ± 10.09 mm, p = 0.887), and suprasellar extension (25.8% vs. 25.5%, p = 1.000).

### 3.3 Operative and early postoperative findings

Multilayer reconstruction was performed in 72 patients (32.4%), and fat graft was used in 54 (24.3%). Postoperative lumbar drainage was performed in 34 patients (15.3%). Diaphragma sellae descent was observed on postoperative imaging in 84 patients (37.8%). Gross total resection (GTR) was achieved in 172 patients (77.5%), while near-total or subtotal resection was achieved in 50 (22.5%).

Postoperatively, hormonal deficiency involving at least one axis was observed in 89 patients (40.1%), including hypocortisolism in 67 (30.2%) and hypothyroidism in 46 (20.7%). The mean length of hospital stay was 2.84 ± 1.29 (range, 2–8) days for the entire cohort.

No significant differences were observed between Groups 1 and 2 in terms of postoperative lumbar drainage (17.5% vs. 12.7%, p = 0.428), diaphragma sellae descent (39.2% vs. 36.3%, p = 0.679), GTR rates (78.3% vs. 76.5%, p = 0.865), and length of hospital stay (2.95 ± 1.43 vs. 2.71 ± 1.10 days, p = 0.395). Preoperative suprasellar extension and postoperative diaphragma sellae descent were evaluated as distinct radiological variables assessed at different time points. Accordingly, the frequencies of these variables were not expected to be identical, and postoperative diaphragma sellae descent could also be observed in patients without preoperative suprasellar extension.

The mean follow-up duration for the entire cohort was 10.1 ± 3.6 (range, 4–16) months.

### 3.4 Endocrine, biochemical, and pathological outcomes

The pre- and postoperative biochemical and pathological findings of the entire cohort are presented in **Table 1**, and some comparative findings between Groups 1 and 2 are presented in **Table 2**.

Mean preoperative sodium levels were 139.47 ± 2.27 (range, 134–145) mEq/L, and sodium levels on postoperative day 1 were 138.44 ± 2.24 (range, 130–144) mEq/L. Mean preoperative-to-postoperative change in sodium levels was 1.91 ± 1.52 (range, 0–8) mEq/L. Sodium levels changed by <3 mEq/L in 166 patients (74.8%) and by ≥3 mEq/L in 56 patients (25.2%). Mean pre- and postoperative glucose levels were 102.86 ± 17.55 (range, 80–150) mg/dL and 121.36 ± 24.87 (range, 79–200) mg/dL. Mean pre- and postoperative potassium levels were 4.29 ± 0.36 (range, 3.5–5.2) mEq/L and 4.18 ± 0.47 (range, 3.2–5.4) mEq/L.

Pathological distribution analysis revealed nonsecretory adenomas in 100 patients (45.0%), growth hormone–secreting adenomas in 52 (23.4%), prolactinomas in 37 (16.7%), adrenocorticotropic hormone–secreting adenomas in 26 (11.7%), apoplexy in 6 (2.7%), and thyroid-stimulating hormone–secreting adenoma in 1 patient (0.5%). The mean Ki-67 index was 2.64 ± 1.83 (range, 1–12).

No significant differences were observed between Groups 1 and 2 in terms of preoperative-to-postoperative change in sodium level ≥ 3 mEq/L (26.7% vs. 23.5%, p = 0.643), postoperative hypokalemia (15.8% vs. 10.8%, p = 0.368), and Ki-67 index ≥ 3% (34.2% vs. 32.4%, p = 0.886).

Regarding tumor characteristics, 159 patients (71.6%) had soft tumors and 63 (28.4%) had firm tumors.

Regarding remission status (available for 116 patients who had secretory adenomas), 99/116 patients (85.3%) achieved remission postoperatively.

### 3.5 Characteristics of postoperative delayed hyponatremia

A total of 30 patients (13.5%) developed delayed hyponatremia after EETS in our cohort, including 23 of 120 patients (19.2%) from Group 1 and 7 of 102 patients (6.9%) from Group 2. A significant difference (p = 0.013) was observed in the group-wise proportions of patients developing delayed postoperative hyponatremia. The reduction in delayed postoperative hyponatremia from 19.2% in the fluid unrestricted group to 6.9% in the fluid-restricted group corresponded to an absolute risk reduction of 12.3 percentage points and an estimated number needed to treat of 9. Thus, approximately nine patients would need to be managed under the fluid-restriction protocol to prevent one additional case of delayed postoperative hyponatremia (**Table 2**).

The characteristics of patients who developed delayed postoperative hyponatremia in Groups 1 and 2 are presented in **Table 2**.

Mean sodium level was 124.8 ± 5.3 (range, 112–134) mEq/L, and mean time to onset was 7.87 ± 1.68 (range, 6–12) days postoperatively. Of the 30 cases of hyponatremia, 28 were symptomatic. The most frequently observed symptom was gastrointestinal complaints (n = 17, 60.7%), particularly nausea (n = 12) and vomiting (n = 16), followed by neurological and general symptoms such as headache (n = 7), dizziness (n = 5), and fatigue (n = 1). Less commonly, patients reported abdominal discomfort, including distension (n = 1), and diarrhea (n = 1). Some patients experienced two or more symptoms. Of the 30 patients who developed delayed hyponatremia, the condition was mild (sodium: 131–135 mEq/L) in 2 patients (6.7%), moderate (sodium: 126–130 mEq/L) in 17 (56.7%), and severe (sodium ≤ 125 mEq/L) in 11 (36.7%). A total of 28 of the 30 patients required hospital readmission, and mean length of hospital stay after readmission was 4.64 ± 1.26 (range, 3–8 days).

All patients in Group 2 (fluid-restricted group) who developed delayed postoperative hyponatremia were readmitted to the hospital and managed with close clinical and biochemical monitoring. By contrast, two patients in Group 1 who developed mild hyponatremia (serum sodium levels of 132 and 134 mmol/L) were managed conservatively on an outpatient basis. Both patients remained clinically stable and were closely followed with serial sodium measurements without requiring hospital readmission or escalation of care. Patients hospitalized due to delayed postoperative hyponatremia were managed on the ward with fluid restriction (500 mL/day) and close sodium monitoring. Hypertonic saline therapy was initiated in patients whose symptoms or sodium levels did not improve despite fluid restriction. Patients were discharged after symptom resolution and normalization of serum sodium levels.

Of the patients who developed delayed postoperative hyponatremia, only one—a 43-year-old woman with Cushing’s disease in the non–fluid-restricted group—presented with delayed postoperative hyponatremia 6 days after discharge and had a serum sodium level of 112 mEq/L at readmission. The patient had achieved postoperative biochemical remission and was receiving glucocorticoid replacement therapy according to the institutional endocrine protocol. Therefore, untreated adrenal insufficiency was considered unlikely to represent the primary cause of the delayed postoperative hyponatremia. Due to persistent symptoms, comorbidities, and lack of improvement despite fluid restriction and hypertonic saline therapy on the ward, she was admitted to the intensive care unit for close monitoring because of the risk of cerebral edema. Her serum sodium level gradually normalized with hypertonic saline in addition to continued fluid restriction. She remained in the intensive care unit for 3 days, followed by 4 days of observation on the neurosurgical ward, and was subsequently discharged in stable condition.

In our series, none of the patients who developed delayed postoperative hyponatremia experienced permanent complications related to hyponatremia, and no mortality was observed. Additionally, among the 102 patients managed under the fluid-restriction protocol, no patient developed hypernatremia, acute kidney injury or clinically significant dehydration or hypovolemia requiring intravenous fluid administration, urgent evaluation, hospital readmission, or early termination of the protocol. No patient required discontinuation or liberalization of fluid restriction because of intolerable thirst.

### 3.6 Analysis of postoperative delayed hyponatremia

**Table 1** summarizes the comparison of factors between patients diagnosed with and without delayed postoperative hyponatremia.

No significant differences were observed between patients who developed delayed postoperative hyponatremia and those who did not in terms of female sex (60.0% vs. 55.7%, p = 0.810), surgical history (30.0% vs. 24.0%, p = 0.627), age (p = 0.963), BMI (p = 0.446), maximum tumor diameter (p = 0.505), adenoma type (secretory vs. nonsecretory, p = 0.267), Ki-67 index (p = 0.813), and biochemical remission status (p = 0.735).

Preoperative imaging revealed that suprasellar extension was more frequent in patients with delayed postoperative hyponatremia (56.7% vs. 20.8%, p = 0.01).

The proportion of patients with Knosp grade 3 or grade 4 tumors was 16.7% (n = 5/30) in the delayed postoperative hyponatremia group and 26.6% (n = 51/192) in the non- delayed postoperative hyponatremia group, with the difference being nonsignificant (p = 0.350).

The prevalence of preoperative hormone deficiency was 53.3% (n = 16/30) in the delayed postoperative hyponatremia group and 37.5% (n = 72/192) in the non- delayed postoperative hyponatremia group, but this difference was nonsignificant (p = 0.148). By contrast, postoperative pituitary hormone deficiency was observed in 70.0% (n = 21/30) of patients in the delayed postoperative hyponatremia group, compared with 35.4% (n = 68/192) in the non- delayed postoperative hyponatremia group, representing a significant difference (p = 0.001). The two groups did not differ significantly in terms of preoperative hypocortisolism (p = 0.969), but preoperative hypothyroidism was significantly more prevalent in the delayed postoperative hyponatremia group compared with the non- delayed postoperative hyponatremia group (26.7% vs. 10.4%, p = 0.033). The delayed postoperative hyponatremia group exhibited significantly lower preoperative sodium levels and postoperative day 1 sodium levels than the non- delayed postoperative hyponatremia group (preoperative: 138.60 ± 1.38 [137–142] mEq/L vs. 139.60 ± 2.35 [134–145] mEq/L, p = 0.005; postoperative day 1: 135.37 ± 2.72 [130–139] mEq/L vs. 138.92 ± 1.72 [135–144] mEq/L, p < 0.001). A significantly higher proportion of patients in the delayed postoperative hyponatremia group showed a preoperative-to-postoperative sodium change ≥ 3 mEq/L (78.6% vs. 50.0%), whereas a lower proportion of patients showed a sodium change < 3 mEq/L (21.4% vs. 50.0%, p = 0.002).

The two groups did not differ significantly in pre- and postoperative day 1 glucose and pre- and postoperative day 1 potassium (p = 0.946, 0.208, 0.202, and 0.089, respectively).

Postoperative hypokalemia was significantly more prevalent in the delayed postoperative hyponatremia group (40.0% vs. 9.4%, p < 0.001).

Postoperative imaging after tumor resection revealed that diaphragmatic descent was significantly more frequent in the delayed postoperative hyponatremia group (73.3% vs. 32.3%, p < 0.001).

A significantly higher proportion of patients in the delayed postoperative hyponatremia group developed firm tumors (63.3% vs. 22.9%, p < 0.001), indicating that firm tumors were strongly associated with delayed postoperative hyponatremia development.

Postoperative lumbar drainage was performed in 50.0% of patients who developed delayed postoperative hyponatremia, compared with 9.9% of patients in the non–delayed postoperative hyponatremia group (p < 0.001).

GTR was achieved in 86.7% of patients who developed delayed postoperative hyponatremia, compared with 76.0% of patients who did not, and this difference was nonsignificant (p = 0.289).

Hospital stay was significantly longer in the delayed postoperative hyponatremia group (4.63 [2–8] days vs. 2.56 [2–6] days, p = 0.001).

### 3.7 Logistic regression and ROC curve analysis of postoperative delayed hyponatremia

A logistic regression analysis was performed to determine the factors affecting delayed postoperative hyponatremia. The statistically significant parameters in the univariate analyses were included in the multiple logistic regression model.

Logistic regression revealed that preoperative comorbidities, postoperative diaphragma sellae descent, postoperative hypothyroidism, postoperative hypokalemia, and longer hospital stay were independently associated with delayed postoperative hyponatremia (p = 0.002, 0.029, 0.011, 0.002, and <0.001, respectively; **Table 3**).

**Table 3:** Logistic regression analysis to determine the factors affecting postoperative delayed hyponatremia. OR, odds ratio; CI, confidence interval.

	Multivariate Analysis		
**Values**	**OR**	**95% CI**	**P**
**Group**			
Group 1	3.525	(0.908–13.687)	0.069
Group 2	1.0 (Reference)	1.0 (Reference)	
Comorbidities			
Yes	9.915	(2.377–41.353)	**0.002**
No	1.0 (Reference)	1.0 (Reference)	
Preoperative–Postoperative Sodium Change Value (mEq/L)			
<3 (mEq/L)	1.0 (Reference)	1.0 (Reference)	
≥3 (mEq/L)	3.108	(0.727–13.294)	0.126
Suprasellar Extension of Tumor			
Yes	1.427	(0.339–6.018)	0.628
No	1.0 (Reference)	1.0 (Reference)	
Diaphragma Sella Descent			
Yes	4.553	(1.167–17.773)	**0.029**
No	1.00 (Reference)	1.0 (Reference)	
Postoperative Hormonal Deficiency			
Hypocortisolism – Yes	2.407	(0.577–10.040)	0.228
Hypocortisolism – No	1.0 (Reference)	1.0 (Reference)	
Hypothyroidism – Yes	6.417	(1.527–26.962)	**0.011**
Hypothyroidism – No	1.0 (Reference)	1.0 (Reference)	
Postoperative Hypokalemia			
Yes	8.114	(2.186–30.127)	**0.002**
No	1.0 (Reference)	1.0 (Reference)	
**Length of Hospital Stay (days)**	2.724	(1.925–3.854)	**<0.001**

**Figure 1:**
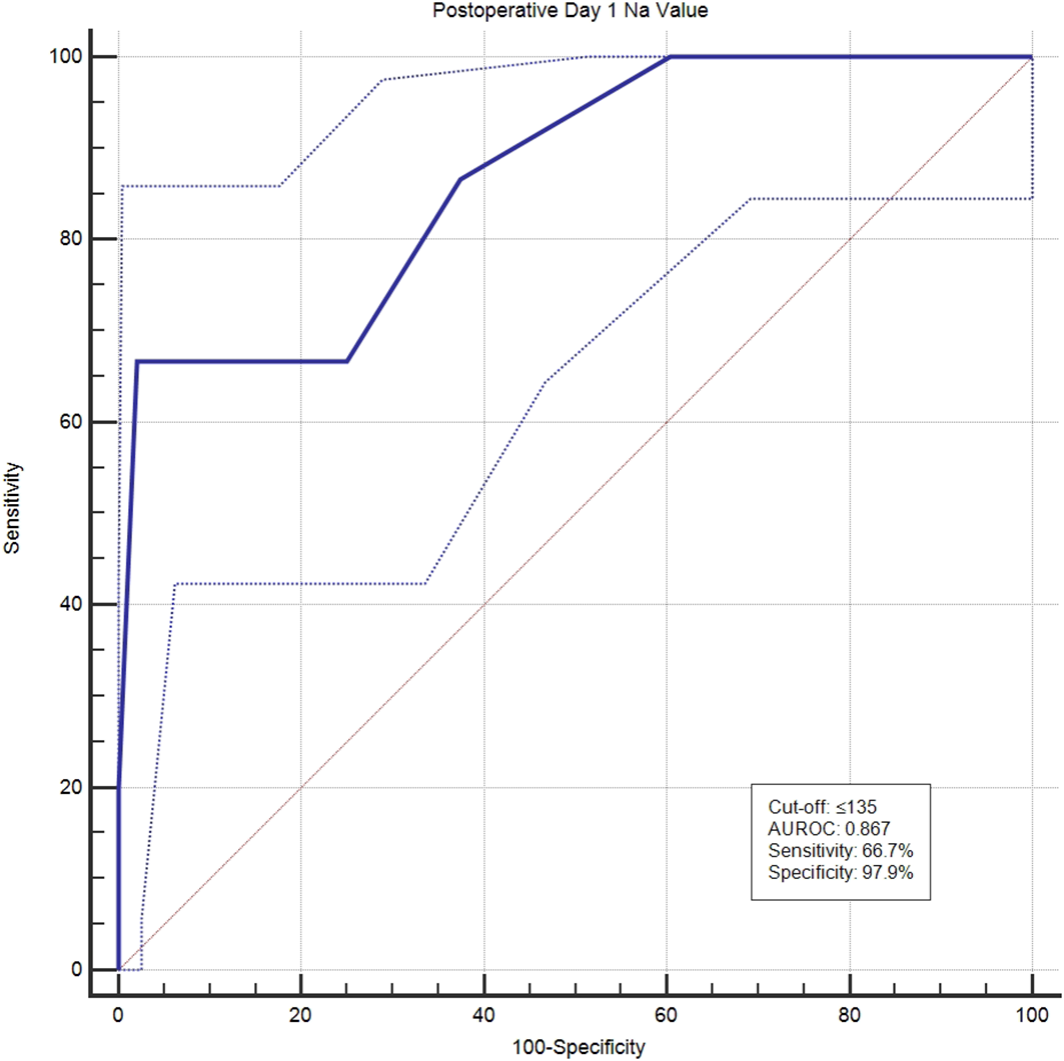
ROC curve analysis of postoperative day 1 serum sodium levels.

In addition, we performed ROC curve analysis on postoperative day 1 serum sodium level. The results showed that postoperative day 1 serum sodium level had good discriminative ability for predicting delayed postoperative hyponatremia (area under the curve: 0.867, 95% confidence interval: 0.816–0.909, p < 0.001). At the optimal cut-off value (≤135), it demonstrated high specificity (97.9%) but moderate sensitivity (66.7%; **Figure 1**).

## 4 Discussion

Postoperative hyponatremia after EETS is primarily related to disturbances in water–sodium balance, often occurring in the context of postoperative DI followed by delayed hyponatremia (2–8, 25). A triphasic pattern of polyuria–oliguria–polyuria has been described following hypothalamic–pituitary surgery, with the oliguria phase contributing to hyponatremia when fluid intake is not appropriately reduced (26, 27). SIADH is considered the principal pathophysiological mechanism underlying delayed postoperative hyponatremia, arising from disruption of the hypothalamic–neurohypophyseal axis and resulting in dysregulated ADH secretion and dilutional hyponatremia. However, because complete diagnostic criteria for SIADH were not consistently available in this retrospective cohort, the primary outcome of the present study was delayed postoperative hyponatremia rather than confirmed SIADH.

Physiologically, ADH release is primarily regulated by plasma osmolality, and it promotes renal water reabsorption via V2 receptors and aquaporin-2 channels (28). Postoperative factors such as pituitary gland and stalk manipulation, perioperative fluid management, transient DI, autonomic alterations, and secondary adrenal insufficiency may further contribute to this dysregulation (4, 5, 21, 23, 29, 30). Clinically, delayed postoperative hyponatremia typically manifests within the first postoperative week, with symptom severity depending on the degree of hyponatremia. Despite these proposed mechanisms, the exact pathophysiology of delayed hyponatremia remains incompletely understood.

Delayed hyponatremia is a leading cause of hospital readmission after pituitary surgery (9, 31). Consistent with this, patients who developed delayed postoperative hyponatremia in our cohort had significantly longer hospital stays. Moreover, length of stay emerged as an independent predictor (p < 0.001) of delayed postoperative hyponatremia in logistic regression analysis, likely reflecting the clinical severity and impact of the condition.

Delayed hyponatremia is a well-recognized postoperative complication of EETS. Reported incidence rates vary considerably in the literature, largely depending on patient characteristics, diagnostic criteria, and postoperative management strategies (1–9, 11, 13, 20, 32). Fluid restriction has increasingly been emphasized as both a preventive and therapeutic approach.

Fluid restriction mitigates ADH-mediated water retention and limits the development of dilutional hyponatremia. Fluid restriction has consistently demonstrated the capacity to reduce the incidence of delayed postoperative hyponatremia across several studies (22–24, 30). In line with this evidence, our results also demonstrated a considerable protective effect of fluid restriction, with the fluid-restricted group exhibiting a markedly lower delayed postoperative hyponatremia incidence rate than the non–fluid-restricted group (6.9% vs. 19.2%, p = 0.013). However, unlike some reports indicating near-complete prevention of delayed postoperative hyponatremia -related readmissions following fluid restriction (22), clinically significant cases requiring hospitalization were still observed in our cohort, suggesting that while fluid restriction is effective, it does not eliminate the risk of delayed postoperative hyponatremia. Our findings are also consistent with current international clinical practice guidelines, which recommend fluid restriction as the first-line treatment for euvolemic hyponatremia caused by SIADH in the absence of severe symptoms (18, 21, 33, 34). Nevertheless, because complete diagnostic criteria for SIADH were not available for all patients, the observed benefit of fluid restriction should be interpreted as a reduction in delayed postoperative hyponatremia rather than as direct evidence that all cases represented confirmed SIADH.

However, the optimal volume and duration of prophylactic fluid restriction remain uncertain. Previous protocols have varied substantially. Fixed 1.0-L/day regimens have been used for 1 week after discharge or during postoperative days 4–8 and have reduced postoperative SIADH or hyponatremia in several cohorts (20, 22, 35), whereas Deaver et al. used 1.5 L/day for 2 weeks together with postoperative sodium testing and reported fewer hyponatremia-related readmissions (23). A systematic review reported fluid-restriction targets ranging from 1.0 to 2.5 L/day and durations extending from postoperative days 1 to 15 (36). More recently, a randomized trial compared moderate restriction—1.8 L/day, or 2.0 L/day for patients weighing >100 kg—with strict restriction—1.0 L/day, or 1.2 L/day for patients weighing >100 kg—from postoperative days 3 to 14; severe hyponatremia occurred in 0% of the strict-restriction group, compared with 5.1% and 7.4% in the moderate-restriction and ad-lib groups, respectively (37). Thus, the 1000-mL/day limit in our study should be interpreted as a pragmatic fixed-volume institutional protocol aligned with the stricter published regimens, rather than as evidence that 1000 mL/day is universally superior to 1200 or 1500 mL/day or to a weight-adjusted strategy. Although recent data support stricter restriction, the optimal balance among efficacy, tolerability, duration, and patient-specific adjustment remains unresolved.

Early identification of hyponatremia is essential to prevent further electrolyte imbalance and associated complications. Consistent with previous reports (25, 38, 39), our findings demonstrate an association between early and delayed hyponatremia, suggesting that a subset of patients is predisposed to prolonged disturbances in sodium homeostasis. This vulnerability may be related to varying degrees of injury to the posterior pituitary stalk or hypothalamic–neurohypophyseal pathways during surgery, resulting in delayed normalization of ADH regulation. In our cohort, preoperative sodium levels, early postoperative sodium levels, and early sodium decline of ≥3 mEq/L emerged as significant predictors of delayed postoperative hyponatremia (p = 0.005, <0.001, and 0.002, respectively). These findings suggest that early sodium changes may serve as reliable predictors of delayed postoperative hyponatremia risk, underscoring the importance of close perioperative electrolyte monitoring.

Delayed hyponatremia following EETS is primarily attributed to SIADH, typically emerging several days postoperatively as a delayed neuroendocrine response rather than an immediate disturbance. Surgical injury or traction on the pituitary stalk may contribute to this process, and delayed recovery of pituitary function may exacerbate ADH dysregulation (40–42). In the present study, delayed hyponatremia occurred typically around postoperative day 7.9, supporting the concept of a delayed neuroendocrine mechanism rather than an immediate postoperative disturbance.

DI is a common complication after EETS, characterized by ADH deficiency and free water loss leading to hypernatremia (22, 29, 43–45). Although usually transient, pituitary stalk injury may result in persistent dysfunction (22). Desmopressin is the gold standard treatment, but excessive use may cause iatrogenic hyponatremia, and intraoperative administration has also been associated with early sodium disturbances (29, 46). Previous studies have demonstrated a link between DI and delayed hyponatremia, likely due to unregulated vasopressin release following neurohypophyseal injury, resulting in delayed postoperative hyponatremia (41, 42). According to the literature, this biphasic response has been associated with a significantly increased risk of delayed hyponatremia (p = 0.001) (14, 40). To avoid this confounding effect, patients with preoperative DI or those requiring early postoperative desmopressin were excluded from our study, allowing a more accurate assessment of independent predictors of delayed postoperative hyponatremia.

Although patients with preoperative diabetes insipidus and those requiring early postoperative desmopressin therapy were excluded to minimize the inclusion of patients with triphasic water-balance disorders, triphasic response cannot be diagnosed solely on the basis of urine output, because urine output during the antidiuretic/SIADH-like phase may be normal or low. Rather, diagnosis requires a characteristic sequential clinical and biochemical pattern consisting of an initial diabetes insipidus/arginine vasopressin deficiency phase, followed by an antidiuretic hyponatremic phase, and finally recurrent diabetes insipidus. Because serial urine osmolality, urine sodium concentration, and complete fluid-balance data were not consistently available for all patients, retrospective identification of every triphasic response was not possible. Therefore, isolated delayed postoperative hyponatremia and delayed hyponatremia occurring as the antidiuretic phase of a triphasic response could not be definitively distinguished in every case. Nevertheless, the mean onset of delayed postoperative hyponatremia in our cohort was postoperative day 7.9, which is consistent with the typical timing of isolated delayed postoperative hyponatremia after pituitary surgery, usually around postoperative days 6–8. Recognizing this distinction is clinically important because isolated delayed postoperative hyponatremia, most commonly attributed to SIADH, differs from triphasic-response-associated hyponatremia in both pathophysiology and prognosis. In particular, a triphasic response may indicate more extensive hypothalamic–neurohypophyseal injury and may carry a higher risk of persistent diabetes insipidus (14, 21, 40).

Age has been suggested as a potential risk factor for delayed hyponatremia due to altered ADH regulation and reduced physiological reserve in older adults (2, 16, 47). Older individuals may exhibit an exaggerated ADH response to osmotic stimuli, increasing susceptibility to hyponatremia. Similarly, sex-related differences have been observed, with some studies reporting a higher risk in women, possibly related to hormonal influences such as estrogen-mediated modulation of ADH pathways (4, 6, 29, 47). However, in our cohort, neither age (p = 0.963) nor sex (p = 0.810) was significantly associated with delayed postoperative hyponatremia development. These findings suggest that in the context of contemporary surgical practice, perioperative and surgical factors may play a more dominant role than demographic characteristics in determining delayed postoperative hyponatremia risk.

Comorbidities such as renal disease, hypertension, and diabetes mellitus can also influence hyponatremia onset through their effects on fluid balance and osmotic regulation (48, 49). In our study, the use of medical treatment for at least one preoperative comorbidity was identified as an independent predictor of delayed postoperative hyponatremia development (p < 0.001), suggesting that baseline physiological reserve and systemic homeostasis may play a key role in the regulation of postoperative sodium balance. Lower BMI has also been reported as a potential risk factor for delayed postoperative hyponatremia, possibly due to differences in total body water distribution (2, 6, 24, 35). However, in our analysis, BMI was not significantly associated with delayed postoperative hyponatremia development (p = 0.446), indicating that its impact may be limited or influenced by other perioperative factors.

Tumor-related anatomical and surgical factors have been suggested to influence the development of postoperative hyponatremia. Larger tumors are generally considered to increase hyponatremia risk due to greater manipulation of the pituitary gland and stalk (2, 3, 7, 50). However, in our cohort, tumor size was not an independent predictor of delayed postoperative hyponatremia (p = 0.505), indicating that size alone may be insufficient without additional contributing factors such as anatomical extension or postoperative changes. By contrast, our findings demonstrated that both suprasellar extension and diaphragma sellae descent were significantly associated with delayed postoperative hyponatremia development (p = 0.01 and p < 0.001, respectively). Suprasellar extension likely increases the degree of hypothalamic and stalk manipulation, thereby predisposing patients to ADH dysregulation. However, because suprasellar extension was recorded as a binary variable in this retrospective study, this finding should be interpreted cautiously, as we could not distinguish mild suprasellar extension abutting the optic chiasm from more extensive superior extension toward the third ventricle or foramen of Monro. Similarly, diaphragmatic descent may reflect postoperative decompression and traction on the pituitary stalk, further contributing to abnormal ADH secretion. These results highlight the importance of tumor location and postoperative structural changes rather than tumor size alone. Although higher Knosp grades are associated with increased surgical complexity and potential stalk manipulation (2), Knosp grade was not significantly associated with delayed postoperative hyponatremia in our cohort (p = 0.350). This suggests that anatomical factors directly affecting the hypothalamic–pituitary axis may play a more critical role than overall surgical difficulty in the development of postoperative hyponatremia. These variables should not be interpreted as representing the same anatomical phenomenon. Whereas suprasellar extension reflects preoperative tumor growth beyond the diaphragma sellae, diaphragma sellae descent represents a postoperative anatomical change following tumor removal.

Although more extensive resections may theoretically increase the risk of pituitary stalk injury, previous studies have reported inconsistent findings regarding this association (27, 51). In line with these observations, gross-total resection was not associated with delayed postoperative hyponatremia in our cohort, suggesting that the extent of resection alone may not be a decisive determinant of postoperative sodium imbalance. Postoperative lumbar drainage was more frequently observed in patients who developed delayed postoperative hyponatremia. However, this association should not be interpreted as causal. Lumbar drainage itself is unlikely to contribute directly to the pathogenesis of delayed postoperative hyponatremia. Rather, it is typically used in patients with intraoperative cerebrospinal fluid leakage, larger or more complex tumors, and more extensive skull base reconstruction. Therefore, lumbar drainage most likely represents a surrogate marker of greater surgical complexity and more extensive manipulation or displacement of the pituitary stalk and hypothalamic–diencephalic region after tumor resection, rather than an independent risk factor for delayed postoperative hyponatremia.

Hypopituitarism, particularly hypocortisolemia and hypothyroidism, can contribute to hyponatremia through impaired free water clearance (8, 40, 45). In our study, the postoperative hypothyroidism variable was based on a biochemical thyroid profile compatible with central hypothyroidism obtained on postoperative day 1. Because delayed postoperative hyponatremia was defined as occurring after postoperative day 3, the thyroid abnormality preceded the qualifying sodium decline. Thus, the association was not based on thyroid dysfunction detected as a consequence of the subsequent hyponatremic episode.

Importantly, this variable should not be interpreted as representing a uniformly untreated or inadequately replaced population. Patients not receiving levothyroxine preoperatively underwent additional endocrine evaluation, after which thyroid hormone replacement was prescribed when indicated. In patients already receiving levothyroxine, treatment was reassessed and the dose was modified when necessary. Nevertheless, the exact timing of levothyroxine initiation, dose adjustment, and biochemical response relative to the sodium decline was not analyzed as a time-dependent exposure. Therefore, the present findings do not demonstrate that untreated or inadequately replaced hypothyroidism directly caused delayed postoperative hyponatremia. Early postoperative thyroid-axis dysfunction may contribute to impaired water excretion, but it may also serve as a marker of broader postoperative pituitary dysfunction.

Management strategies are largely determined by the severity and symptomatology of hyponatremia. Mild cases may be managed conservatively with fluid restriction and oral sodium supplementation, whereas moderate-to-severe or symptomatic cases require hospitalization and close biochemical monitoring (33), with treatment options including intravenous saline, hypertonic saline, and, in selected patients, pharmacological agents such as tolvaptan (34). Careful correction is essential to avoid complications such as cerebral edema or osmotic demyelination syndrome.

In our cohort, all patients who developed delayed postoperative hyponatremia in the fluid-restricted group were hospitalized and managed with close clinical and biochemical monitoring, whereas selected mild cases in the non–fluid-restricted group were successfully followed on an outpatient basis. Management followed a stepwise, severity-driven approach, including strict fluid restriction and serial sodium monitoring, with escalation to hypertonic saline in patients who did not demonstrate adequate clinical or biochemical improvement (33, 34). Only one patient required intensive care unit admission due to severe hyponatremia and persistent symptoms, and she was successfully treated with gradual sodium correction. The mean time to delayed postoperative hyponatremia onset was 7.87 days, consistent with previous reports, underscoring the importance of continued monitoring beyond the early postoperative period. Consistent with prior findings, most patients presented with moderate-to-severe, symptomatic hyponatremia, predominantly with gastrointestinal manifestations (22–24, 30). The rate of hospital readmission was high (93.3%), in contrast to studies reporting reduced hospitalization through structured outpatient monitoring protocols (22, 23). This discrepancy may reflect differences in institutional thresholds for admission and follow-up strategies, as hospitalization was preferred in our cohort for symptomatic or moderate-to-severe cases to ensure close observation and timely intervention. Patient outcomes were favorable in our cohort, with no permanent neurological complications or mortality, supporting the effectiveness of early recognition and appropriate management.

Overall, postoperative fluid restriction significantly reduced delayed postoperative hyponatremia incidence in our cohort. Given the multifactorial etiology of delayed hyponatremia, early risk stratification appears critical for optimizing perioperative management and improving patient outcomes. Future studies should aim to refine predictive tools and establish standardized fluid-restriction protocols.

This study has some limitations. An important limitation of this study is that complete diagnostic criteria for SIADH—including serum/plasma osmolality, urine osmolality, urine sodium concentration, standardized clinical assessment and documentation of extracellular volume status, dietary sodium intake, confirmation of normal adrenal and thyroid function according to full diagnostic criteria, and documentation of no recent diuretic use—were not consistently available because of the retrospective study design. Although available clinical records were reviewed for findings suggestive of hypovolemia, extracellular volume status was not documented in a standardized manner in all patients. Therefore, although SIADH was considered the most likely underlying mechanism in many patients with delayed postoperative hyponatremia, definitive differentiation from other causes, particularly cerebral salt wasting, could not always be established (6, 15–18). Second, the retrospective study design may introduce selection bias and limit the ability to establish causal relationships. Third, the study was conducted at a single center, which may affect the generalizability of the findings. Additionally, variations in postoperative monitoring and management strategies may have influenced the observed outcomes. Although all patients with pituitary apoplexy underwent routine perioperative endocrine evaluation and received glucocorticoid replacement therapy according to institutional practice, pituitary apoplexy itself may still represent a potential source of residual confounding because of its association with acute hypothalamic–pituitary dysfunction. Furthermore, suprasellar extension was evaluated as a binary radiological variable rather than according to a standardized grading system. Therefore, the potential influence of different degrees of suprasellar extension on delayed postoperative hyponatremia could not be assessed. Finally, because this study evaluated a single fixed-volume protocol, it cannot determine whether 1000 mL/day is superior to 1200 or 1500 mL/day, or whether a weight-adjusted approach would achieve a better balance of efficacy and tolerability. Although BMI was not associated with delayed postoperative hyponatremia in our cohort, this finding should not be interpreted as evidence that body-size adjustment is unnecessary.

A further important limitation is the absence of standardized postdischarge adherence data. Although fluid intake and urine output were monitored during hospitalization, patients did not complete standardized fluid diaries, and adherence after discharge was not systematically quantified or confirmed during follow-up. Thus, group assignment represented exposure to a prescribed institutional protocol rather than verified compliance with the 1000-mL/day target. This may have resulted in exposure misclassification, precluded adherence-stratified analyses, and limits attribution of the observed between-group difference specifically to actual fluid intake. The true efficacy of the restriction among fully adherent patients therefore cannot be determined from the present study.

Despite these limitations, our study has notable strengths. It represents a well-characterized cohort with detailed perioperative clinical, biochemical, and radiological data, allowing a comprehensive evaluation of factors associated with delayed postoperative hyponatremia. Furthermore, the inclusion of a fluid-restricted group provides clinically relevant insight into the preventive role of postoperative fluid management. The identification of both preoperative and early postoperative predictors also highlights the potential for early risk stratification and targeted patient management.

## 5 Conclusion

Delayed postoperative hyponatremia remains a relevant complication following EETS. Our findings suggest that implementation of a routine postoperative fluid-restriction protocol is associated with a markedly lower incidence of delayed postoperative hyponatremia, although the absence of objective postdischarge adherence data limits estimation of the true treatment effect. Early postoperative sodium changes and selected clinical factors may help identify high-risk patients. Therefore, combining fluid restriction with close monitoring and risk-based follow-up is essential to minimize morbidity and improve outcomes.

## Data Availability

The original contributions presented in the study are included in the article/supplementary material. Further inquiries can be directed to the corresponding author.
